# Assessing the daily stability of the cortisol awakening response in a controlled environment

**DOI:** 10.1186/s40359-016-0107-6

**Published:** 2016-01-28

**Authors:** Greg J. Elder, Jason G. Ellis, Nicola L. Barclay, Mark A. Wetherell

**Affiliations:** Biomedical Research Building, Campus for Ageing and Vitality, Institute of Neuroscience, Newcastle University, Newcastle upon Tyne, NE4 5PL UK; Northumbria Centre for Sleep Research, Northumbria University, Newcastle upon Tyne, NE1 8ST UK

**Keywords:** Cortisol awakening response, Sleep, Hypothalamic-pituitary-adrenal axis, Cortisol

## Abstract

**Background:**

Levels of cortisol, the end product of the hypothalamic-pituitary-adrenal (HPA) axis, display a sharp increase immediately upon awakening, known as the cortisol awakening response (CAR). The daily stability of the CAR is potentially influenced by a range of methodological factors, including light exposure, participant adherence, sleep duration and nocturnal awakenings, making inferences about variations in the CAR difficult. The aim of the present study was to determine the daily stability of multiple measurement indices of the CAR in a highly-controlled sleep laboratory environment. A secondary aim was to examine the association between objective sleep continuity and sleep architecture, and the CAR.

**Methods:**

The CAR was assessed in 15 healthy normal sleepers (seven male, eight female, M_age_ = 23.67 ± 3.49 years) on three consecutive weekday mornings. Sleep was measured objectively using polysomnography. Saliva samples were obtained at awakening, +15, +30, +45 and +60 min, from which multiple CAR measurement indices were derived: cortisol levels at each time point, awakening cortisol levels, the mean increase in cortisol levels (MnInc) and total cortisol secretion during the measurement period. Morning 2 and Morning 3 awakening cortisol levels, MnInc and total cortisol secretion were compared and the relationship between Night 1 and Night 2 objective measures of sleep continuity and architecture, and the subsequent CAR, was also assessed.

**Results:**

There were no differences in cortisol levels at each time point, or total cortisol secretion during the CAR period, between Morning 2 and Morning 3. Awakening cortisol levels were lower, and the MnInc was higher, on Morning 3. Morning 2 and Morning 3 awakening levels (*r* = 0.77) and total cortisol secretion (*r* = 0.82), but not the magnitude of increase, were positively associated.

**Conclusions:**

The stability of the CAR profile and total cortisol secretion, but not awakening cortisol levels or the magnitude of increase, was demonstrated across two consecutive mornings of measurement in a highly-controlled environment. Awakening cortisol levels, and the magnitude of increase, may be sensitive to differences in daily activities.

**Electronic supplementary material:**

The online version of this article (doi:10.1186/s40359-016-0107-6) contains supplementary material, which is available to authorized users.

## Background

The stress hormone cortisol is the end product of the hypothalamic-pituitary-adrenal (HPA) axis, a system which aids the adjustment and adaptation to bodily and environmental challenges [1, 2]. This system is under the overall co-ordination of the suprachiasmatic nucleus (SCN), which is the body’s central pacemaker [3]. Cortisol secretion follows a diurnal pattern with a sharp increase in the first hour following awakening, which is known as the cortisol awakening response (CAR). During the CAR period, cortisol levels increase by 38–75 %, peaking approximately 30–45 min post-awakening [1, 4]. Multiple measurement indices can be used to assess the CAR, including cortisol levels at specified time points (e.g. immediately upon awakening), the magnitude of increase in cortisol levels, and total cortisol secretion during the CAR measurement period [4].

It is estimated that 73–77 % of healthy adults display a typical CAR [5], although the exact function of the CAR is still not known [6]. It has been speculated that the CAR may help promote arousal upon awakening, or assist in the recovery from previous experiences [7–9]. It has also been suggested that the CAR is a marker of anticipation; specifically reflecting the preparation for the forthcoming demands of a particular day [1, 10]. Despite the widespread use of the CAR as a comparative marker of HPA axis function within a diverse range of populations [11–14], little is known about the daily stability of the CAR. The CAR shows a great degree of variability when measured between days, meaning that the CAR of a single day appears to be largely affected by situational factors, such as mood or light levels, rather than trait factors. Due to this, multiple measurement days are needed in order to reliably assess the CAR [15].

To date, only one study has measured the CAR in a sleep laboratory environment in normal healthy sleepers, where sleep was not disrupted or manipulated [16] and there are no studies which have examined the CAR over consecutive days in a sleep laboratory environment. Of the other studies which have examined the CAR in a sleep laboratory, the aim has been to examine the subsequent CAR following an experimental manipulation [e.g. 17]. Although Hellhammer and colleagues recommend the collection of the CAR over multiple days of measurement, this is based on ambulatory CAR data [15]. The majority of studies which measure the CAR have done so in an ambulatory environment. However, these studies can be influenced by a range of methodological factors, potentially resulting in misleading or erroneous results.

Firstly, ambulatory studies typically require unsupervised participants to self-collect samples, and poor levels of adherence to sampling protocols can dramatically increase measurement error [4]. This issue was highlighted in one study which tracked sampling times using time-stamped saliva collection bottles, which observed an adherence rate of 74 % [18]. Importantly, participant non-adherence had the greatest impact upon the resulting CAR profile, and the majority (82 %) of non-adherent participants failed to collect two or more samples at required time points [18]. Non-adherence to the awakening sample is particularly problematic, as a delayed awakening sample can flatten the peak, relative to awakening cortisol levels, and thus mimic a deficiency [19, 20]. The potential for poor adherence is therefore one of the main limitations of ambulatory CAR measurement and can be overcome by measuring the CAR in a supervised, laboratory environment.

Secondly, ambulatory CAR studies are also likely to be influenced by intra-individual differences in environmental light exposure, either prior to or during the CAR measurement period. This is of importance to the CAR, since the SCN, which is sensitive to light, co-ordinates the HPA axis [3]; thus, light levels are likely to influence the resulting CAR. The influence of light upon various measurement indices of the CAR has been confirmed by several experimental studies [7, 21, 22].

Thirdly, sleep may also affect the daily stability of the CAR, as sleep duration, the occurrence and duration of nocturnal awakenings, and the time of awakening are all likely to influence the CAR [23]. In order to account for these factors, a highly controlled and consistent measurement environment is needed, across multiple days of measurement. In ambulatory studies participants are generally unsupervised overnight prior to the collection of the CAR. Therefore, nocturnal awakenings may influence the CAR, although these data are generally not collected, or are self-reported. Although actigraphy, which provides objective information regarding sleep continuity, has been employed in ambulatory studies, this has mainly been used to assess whether self-reported awakening times match objective awakening times [24, 25]. A further limitation of actigraphy is that despite the ability to provide more detailed sleep information, this cannot prevent the resulting CAR being influenced by intra-individual differences in sleep architecture [23].

The basic relationship between objective sleep continuity and architecture and the CAR in healthy normal sleepers is currently unclear, as a previous study did not directly examine this relationship in healthy individuals in a sleep laboratory environment [16], and inconsistent findings have previously been observed in the few studies which have examined clinical populations [23]. For example, a study of army veterans with post-traumatic stress disorder did not observe a relationship between sleep architecture and total plasma cortisol secretion during the CAR period [26]. Further, in a sample of alcohol-dependent inpatients, a negative association between the duration of rapid eye movement (REM) sleep and awakening cortisol levels was observed [27]. In a study combining dementia caregivers and non-caregivers, the percentages of sleep spent in stage 1, stage 3 and REM were negatively related to overall awakening cortisol levels, however, it is likely that these results were confounded by between-group differences [28]. As the relationship between objective sleep measures and the CAR is unclear in healthy, normal sleepers, this should first be investigated in a highly-controlled manner before being extended to other populations.

A laboratory environment ensures that sleep duration can be closely monitored, and accounted for if necessary. In the case of sleep duration, the findings are mixed and the relationship between the CAR and sleep duration appears to be influenced by the study design and the choice of CAR measurement indices [5, 29–32]. For example, whilst Kumari and colleagues observed that individuals with a short sleep duration (less than 5 h) displayed a steeper rise in cortisol levels between awakening and +30 min in a large sample of middle-aged adults [29], a meta-analysis indicated that the most consistent association was a positive relationship between sleep duration and awakening cortisol levels [33]. Additionally, little is known about whether differences in the mode of awakening can affect the CAR; on the basis of one single-case study, the CAR did not differ when observed in response to natural awakening, or an awakening caused by an alarm clock [30]. However, a laboratory environment can ensure that the mode of awakening is consistent for all participants (i.e. where all participants have either natural or forced awakenings).

In order to accurately determine whether the CAR is stable, a highly-controlled measurement environment, with the simultaneous monitoring of sleep, is needed to ensure high levels of control over relevant methodological factors. Specifically, a sleep laboratory environment ensures that environmental light levels are standardised prior to and during the CAR measurement period, that other circadian factors including food intake can be taken into account, that nocturnal awakenings are monitored, and that the mode of awakening is consistent between participants, whilst allowing the careful and accurate monitoring of sleep prior to the measurement of the CAR. This environment can also maximise participant adherence by ensuring that the awakening sample is obtained at the appropriate time point, therefore reducing measurement error.

The aim of the present study was to determine the daily stability of multiple measures of the CAR in healthy normal sleepers, with the simultaneous objective monitoring of sleep, within a highly-controlled sleep laboratory environment. A secondary aim of the study was to assess the basic relationship between measures of objective sleep continuity and architecture and the CAR in healthy normal sleepers, given the paucity of research in healthy populations. In order to comprehensively assess the CAR, the CAR was expressed as cortisol levels at each measurement time point, awakening cortisol levels, the mean increase in cortisol levels and total cortisol secretion during the measurement period.

## Methods

### Participants

Eighteen non-smoking healthy normal sleepers (nine male, nine female; M_age_ = 23.46 years, SD_age_ = 3.21 years) were recruited from the staff and student population of Northumbria University using email advertisements. Participants provided written informed consent and were paid £150 upon completion of the study. The study was approved by Northumbria University Faculty of Health Sciences Ethics Committee.

### Procedure

**Fig. 1:**
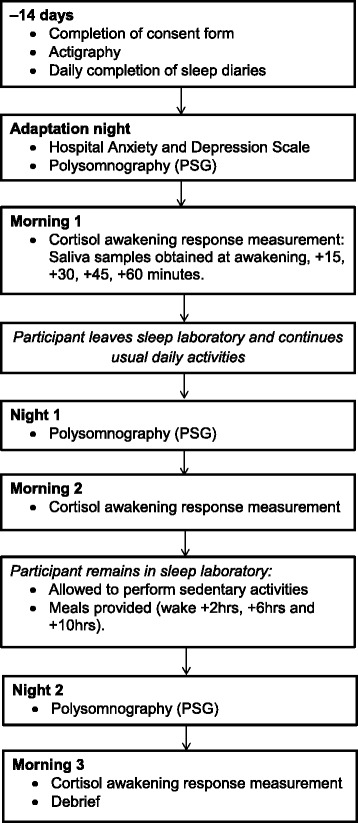
Study procedure

The study procedure is summarised in Figure 1. In order to ensure that participants were healthy good sleepers, all participants were screened for current or previous sleep problems; physical illnesses; shift work; or trans-meridian travel in the three months prior to study enrolment, on the basis of a clinical interview with a member of the research team. In order to determine habitual sleep/wake schedules and verify their stability, participants completed self-reported sleep diaries [34] and wore an actigraph in the two weeks prior to the laboratory stay. Actigraphy data were visually inspected for any evidence of circadian abnormalities before commencing the laboratory study. Participants slept for three consecutive weekday nights in a sleep laboratory (Adaptation Night, Night 1 and Night 2), where sleep was measured objectively using polysomnography (PSG).

The CAR was measured on each of the weekday mornings (Morning 1, Morning 2 and Morning 3), where participants were awoken by a researcher at their scheduled awakening time. Participants were prohibited from eating, drinking (with the exception of a small amount of water), or brushing their teeth, either before or during the measurement period, in order to avoid the potential contamination of saliva samples through abrasion or vascular leakage [4, 35].

Participants left the sleep laboratory approximately one hour after the final saliva sample was obtained on Morning 1 and were instructed to follow their habitual daily routine. Between Night 1 and Morning 3 (a period of approximately 30 h) participants remained in the sleep laboratory, under observation, in order to ensure a stable and consistent environment. Participants were permitted to perform sedentary activities during this period, including reading, watching television or films. During this period, participants were not permitted to leave the laboratory at any point. Standardised meals were provided at identical time points (+2, +6 and +10 h post-awakening) in order to avoid any potential circadian effects of food intake. Participants were debriefed and were allowed to leave the laboratory one hour after the final saliva sample was obtained on Morning 3.

### Cortisol awakening response

The CAR was measured on three consecutive weekday mornings (Morning 1, Morning 2 and Morning 3), where saliva samples were obtained immediately upon awakening, and at +15, +30, +45 and +60 min post-awakening. All saliva samples were collected in the presence of a researcher, who did not engage the participant in conversation during the measurement period. Saliva samples were obtained using Salivettes (Sarstedt, Leicester, UK). To ensure consistency and the collection of sufficient saliva for assaying, all participants were instructed to chew on Salivettes for 60 s.

Saliva samples were stored in a domestic refrigerator immediately following collection, before being frozen at −20 °C at the earliest opportunity, until assaying. Samples were centrifuged at 3000 rpm for 15 min and all assays were performed in-house in order to avoid the potential influence of inter-laboratory analytical variations [36, 37]. All assays were performed using the luminescence immunoassay method, in accordance with manufacturer instructions (Salimetrics, Newmarket, UK; inter-assay coefficients <10 %). Assays were performed in the same laboratory, using identical techniques, in order to avoid bias [36].

### Sleep environment

Participants slept in a windowless room within a sleep laboratory and were awoken by a researcher at their pre-determined awakening time, which was scheduled in accordance with their average weekday bedtime and average awakening time from their baseline sleep diaries. All saliva samples were collected in constant low-intensity ultraviolet light, of approximately one lux, to minimise the influence of light input upon the CAR. Participants were instructed to remain supine in bed during the measurement period.

### Measures

#### Polysomnography

Sleep was monitored objectively using PSG. Recording times were scheduled in accordance with average weekday habitual bedtimes and awakening times (on the basis of baseline two week sleep diary sleep/wake schedules), and did not vary across the laboratory period. Electroencephalogram (EEG) electrodes were placed at FP_1_, FP_2_, F_3_, F_4_, C_3_, C_4_, P_3_, P_4_, O_1_, O_2_ and C_z_, referenced to linked mastoids (M_1_, M_2_) and a ground electrode (FP_z_). PSG also included chin and anterior tibialis electromyogram (EMG), electrooculogram (EOG) and electrocardiogram (ECG) channels, during all recording nights. PSG was recorded using a SOMNOscreen system (SOMNOmedics GmbH, Randersacker, Germany) and impedance levels were maintained below 5kΩ. Recordings were blind-scored in 30-s epochs by an external scorer, where sleep stages were scored in accordance with American Academy of Sleep Medicine guidelines [38].

#### Data analysis

Objective measures of sleep continuity (total sleep time (TST); sleep efficiency (SE%); sleep onset latency (SOL); the number of awakenings (NWAK), wake after sleep onset (WASO)), sleep architecture (percentages of sleep spent in wake, rapid eye movement sleep (REM), stage 1 (N1), stage 2 (N2) and stage 3 (N3); and the latency to each stage of sleep) were derived from PSG data. These measures are described in Table 1.

**Table 1:** Measures of objective sleep continuity and sleep architecture derived from polysomnography data

Measure	Description
Total sleep time (TST)	The number of minutes scored as N1, N2, N3 or REM sleep.
Sleep onset latency (SOL)	The elapsed time from lights out to the first epoch classified as sleep.
Number of awakenings (NWAK)	The number of stage wake occurrences.
Wake after sleep onset (WASO)	Minutes scored as wake from the first epoch of sleep to lights on.
Sleep efficiency (SE%)	Total sleep time (TST) as a percentage of total recording time (TRT) ((TST / TRT x 100) = SE%).
Time in Wake, N1, N2, N3 and REM (%)	Time scored individually as N1, N2, N3 and REM sleep, as a percentage of total sleep time (TST).
REM, N1, N2 and N3 latency (mins)	The elapsed time from lights out to the first epoch of stage REM, N1, N2 and N3 sleep in minutes.

*Abbreviations:* TST: total sleep time, SOL: sleep onset latency, NWAK: number of awakenings, WASO: wake after sleep onset, SE: sleep efficiency, REM: rapid eye movement sleep, N1: stage 1 sleep, N2: stage 2 sleep, N3: stage 3 sleep

The CAR was assessed through the measurement of cortisol levels at each sampling time point (measured in nanomoles per litre (nmol/l), awakening cortisol levels, the mean increase in cortisol levels during the measurement period (MnInc) and total cortisol secretion during the measurement period. The MnInc was derived from the average cortisol levels of all post-awakening samples (measured between +15 and +60 min) [5]. Total cortisol secretion was calculated using the area under the curve with respect to ground (AUC_G_) formula [39] and was expressed in arbitrary units. Shapiro-Wilk tests, conducted on cortisol levels in order to assess normality, were not significant (all *p*-values >0.05) and non-transformed cortisol data were used in all subsequent analyses.

**Fig. 2:**
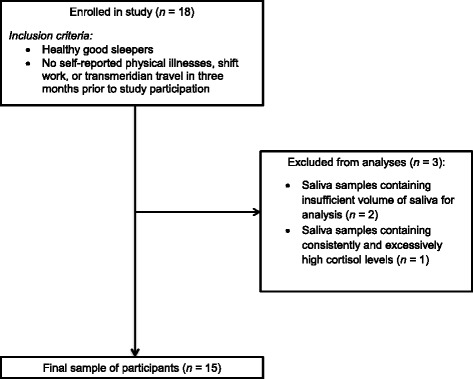
Participant flowchart

PSG data from the Adaptation Night were excluded from further analyses, as PSG alterations are typically observed during the first night of sleep in a laboratory environment [40, 41]. Morning 1 CAR data were also removed for this reason. CAR data from three participants were excluded due to saliva samples containing an insufficient volume of saliva for analysis (*n* = 2) and due to consistently and excessively high cortisol levels [>75 nmol/l; 42] (*n* = 1). This resulted in a final sample of 15 participants (Fig. 2).

A 2 (morning) × 5 (time point) analysis of variance (ANOVA) was conducted in order to compare cortisol levels during the measurement period between Morning 2 and Morning 3, and between each sampling time point. Greenhouse-Geisser adjusted degrees of freedom are reported where appropriate. Effect sizes are reported using partial eta squared (η^2^_p_) values. Paired *t*-tests were used to compare Morning 2 and Morning 3 CAR indices (awakening levels, MnInc and AUC_G_). *Post-hoc* power analyses for these comparisons were calculated using G*Power 3.1 [43].

In order to examine the specific relationship between measures of objective sleep continuity and architecture and CAR indices, Spearman rank correlations were used to examine the association between objective measures of sleep continuity and architecture (TST, SE%, SOL, NWAK, WASO, percentages of sleep spent in N1, N2, N3 and REM, and the latencies to N1, N2, N3 and REM) and CAR measurement indices (awakening levels, MnInc and AUC_G_). This association was examined separately between Night 1 and Morning 2, and between Night 2 and Morning 3. All significance values were adjusted using Bonferroni corrections (*p* = 0.05/39), resulting in an adjusted significance threshold of *p* = 0.0013. Pearson correlations were used to examine the test-retest reliability of the CAR measurement indices between Morning 2 and Morning 3.

## Results

The final sample consisted of 15 healthy sleepers (seven male, eight female, M_age_ = 23.67 years, SD_age_ = 3.49 years) showing normal sleep patterns, as verified by summary PSG data (Table 2).

**Table 2:** Average Night 1 and Night 2 objective sleep measures (*n* = 15)

	Mean	*SD*
TST (mins)	430.12	26.46
SOL (mins)	14.28	11.07
NWAK	13.87	4.89
WASO (mins)	13.12	6.59
SE (%)	94.02	2.77
Time in REM (%)	22.36	3.20
Time in N1 (%)	3.60	1.20
Time in N2 (%)	53.94	5.15
Time in N3 (%)	20.10	5.02
Latency to REM (mins)	105.87	33.79
Latency to N1 (mins)	14.28	11.07
Latency to N2 (mins)	20.73	12.59
Latency to N3 (mins)	33.73	13.77

*Abbreviations:* N1: stage 1 sleep, N2: stage 2 sleep, N3: stage 3 sleep, NWAK: number of awakenings, REM: rapid eye movement sleep, SE: sleep efficiency, SOL: sleep onset latency, TST: total sleep time, WASO: wake after sleep onset

Test-retest correlation results showed significant positive associations between Morning 2 and Morning 3 awakening levels (*r* = 0.77, *p* = 0.001) and total cortisol secretion (AUC_G_: *r* = 0.82, *p* < 0.001). The association between the Morning 2 and Morning 3 CAR MnInc was not significant (*r* = 0.08, *p* > 0.05).

**Fig. 3:**
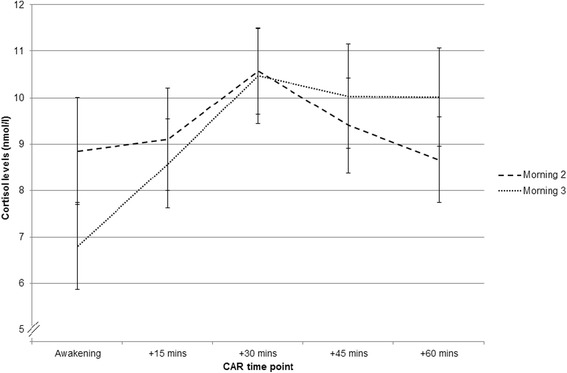
Mean (±SEM) Morning 2 and Morning 3 cortisol levels at each measurement time point (*n* = 15)

As expected, comparisons of cortisol levels at each measurement time point between Morning 2 and Morning 3 showed a significant main effect of time point (*F*(4,56) = 7.44, *p* < 0.001, η^2^_p =_ 0.35), reflecting the typical change in cortisol levels over the CAR measurement period (Fig. 3 & Additional file 1: Table S1). Cortisol levels at each time point did not significantly differ based on the morning of measurement (*F*(1,14) = 0.01, *p* > 0.05, η^2^_p_ = 0.00) and the morning × time point interaction was not significant (*F*(2.84, 39.72) = 2.19, *p* > 0.05, η^2^_p_ = 0.14). Morning 2 and Morning 3 awakening cortisol levels, MnInc and AUC_G_ values are summarised in Table 3. Compared to Morning 2, awakening cortisol levels were significantly lower (*t*(14) = 2.75, *p* < 0.05) and the MnInc was significantly larger (*t*(14) = −2.35, *p* < 0.05) on Morning 3. Total cortisol secretion (AUC_G_) did not significantly differ between Morning 2 and Morning 3 (*t*(14) = 0.16, *p* > 0.05). *Post-hoc* power analyses indicated that the power for these comparisons were 0.83, 0.72 and 0.06 respectively, and that the study had 58 % power to detect medium-sized effects (*d* = 0.50) in these measures.

**Table 3:** Cortisol awakening response measurement indices by morning (*n* = 15)

	Morning 2		Morning 3		M2 vs M3
	Mean	*SD*	Mean	*SD*	*p*-value
Awakening levels (nmol/l)	8.85	4.47	6.80	3.61	0.016
AUC_G_ (arbitrary units)	567.50	219.04	562.35	199.78	0.878
MnInc (nmol/l)	0.58	2.58	2.97	3.20	0.034

*Abbreviations*: AUC_G_ : area under the curve with respect to ground, nmol/l: nanomoles per litre, MnInc: mean increase

The Morning 2 MnInc showed a significant positive association with the percentage of sleep spent in N2 during Night 1 (*r*_s_ = 0.76, *p* < 0.0013). There were no other significant associations between measures of sleep continuity or architecture and CAR measurement indices, either between Night 1 and Morning 2, or Night 2 and Morning 3 (all *p*-values > 0.0013). These are summarised in Additional file 2: Table S2 and Additional file 3: Table S3.

## Discussion

The aim of the current study was to assess the daily stability of multiple measures of the CAR, in a sleep laboratory environment with extremely high levels of control over environmental factors, whilst also accounting for objective measures of sleep. These results indicate that cortisol levels at each sampling time point, and total cortisol secretion, are stable across two consecutive mornings of measurement. However, awakening cortisol levels were lower, and the magnitude of increase was higher, on the second morning of measurement.

The present study also examined the specific relationship between the CAR and objective sleep continuity and architecture in healthy normal sleepers. The results indicated that whilst no objective measures of sleep continuity were associated with the CAR, specific architectural properties of objective sleep during Night 1 were related to the magnitude of the subsequent Morning 2 CAR. This association was not observed between Night 2 sleep and the Morning 3 CAR. Specifically, the percentage of time spent in N2 sleep during Night 1 was positively associated with the magnitude of the subsequent Morning 2 CAR. However, in order to confirm the causal relationship between the percentage of time spent in N2 sleep and the subsequent CAR magnitude, future studies should manipulate sleep architecture by specifically disrupting N2 sleep. This approach will confirm whether changes to sleep architecture can directly affect the subsequent CAR. Due to the modest statistical power of the current study, other potential associations between measures of the CAR and objective sleep continuity and sleep architecture should be examined in a larger sample.

It is possible that the association between objective sleep continuity and architecture is affected by age, as a recent study in school-aged children observed a negative relationship between total cortisol secretion (measured using the AUC_G_) and both sleep duration and the percentage of slow wave sleep, and a positive relationship between the AUC_G_ and N2 sleep [44]. The authors speculate that these results indicate that lower HPA axis activity is associated with more restorative sleep in children. However, this study examined the CAR in an ambulatory environment and the both sleep and the CAR may have been influenced by differences in measurement environment and daily activities. The potential influence of age could be examined further in a laboratory environment.

This study also indicates that both awakening cortisol levels and total cortisol secretion (AUC_G_), but not the MnInc, display high levels of test-retest reliability (*r* values of 0.77, 0.82 and 0.08 respectively). The test-retest reliability of these CAR measures has previously been reported in a large sample of healthy adults (*n* = 509), which observed significant test-retest values between two consecutive days of ambulatory sampling of *r* = 0.37 for awakening cortisol levels, *r* = 0.63 for AUC_G_ values and *r* = 0.47 for MnInc values [5]. The levels of test-retest reliability for awakening cortisol levels and total cortisol secretion are higher in the present study compared to those reported by Wüst and colleagues; potentially due to the reduced influence of sleep, awakening time and light levels prior to and during the CAR measurement period. The present study also indicates that the MnInc does not have a good level of test-retest reliability. Given the highly-controlled measurement environment in the present study, the MnInc may be particularly sensitive to daily activities, since a significantly higher MnInc was observed on Morning 3. Given the potential anticipatory role of the CAR [1, 10, 45], the sensitivity of the CAR to daily activities should be examined further in a laboratory environment.

It is a particular strength of the current study that environmental light levels were standardised, with no intra-individual variability, and were consistent prior to and during the measurement period, as participants were exposed to a consistently low level of light of one lux. This is an advantage over ambulatory studies, and is of particular importance as environmental light can affect various CAR indices [7, 21, 22]. The current study ensured that there was no variation in environmental light levels between participants and that light levels did not vary across each morning of measurement, which cannot be controlled for in ambulatory studies.

Additionally, in the current study, participants remained under observation in the sleep laboratory between Night 1 and Morning 3. Due to the high levels of control, this minimised the influence of other relevant circadian influences upon the CAR. Specifically, between Night 1 and Morning 3, participants were not permitted to exercise, were provided with standardised meals at identical time points relative to their awakening time, and were not permitted to leave the laboratory. This ensured that participants remained in the same environment during the observation period, with no intra-individual variations in food intake, exercise or light exposure, thus minimising the potential circadian influences of these variables [46].

A further strength of the study is that as all saliva samples were obtained in the presence of a researcher, this ensured full participant adherence with the required sampling protocol. In particular, this ensured that the awakening sample, which is especially sensitive to delays in collection, was obtained immediately, therefore minimising the corresponding measurement error [19, 20]. The close monitoring of participants before and during the CAR period also avoided the risk of sample contamination, as participants were not allowed to eat or drink during this period. As the current study employed PSG as a gold-standard method of objective sleep monitoring, this ensured that the effects of objective sleep continuity and architecture upon awakening cortisol levels, the magnitude of increase and total cortisol secretion during the CAR period were accounted for. The use of PSG to monitor participants also ensured that all participants were asleep prior to the awakening sample, and allowed for the potential influence of nocturnal awakenings to be removed.

The main limitation of the present study was in the small sample size. That said, the sample size of the present study is similar to the sample size of other studies where the CAR has been measured in healthy normative individuals in a sleep laboratory environment [16, 17]. Despite this, the study participants were well-characterised and completed a two week period of sleep diaries and actigraphy prior to the laboratory study. In addition, the study results were not influenced by the typical alterations to objective sleep observed during an adaptation night. Whilst future studies may wish to replicate the current findings in a larger sample of participants, the current study accounted for a range of relevant environmental factors which were likely to influence the CAR.

A further limitation of this protocol include the associated costs, and the time-intensive and labour-intensive nature of the study, since a researcher is required to monitor sleep prior to the CAR measurement period and to supervise all saliva sampling. However, these potential limitations are more than outweighed by the extremely high levels of control afforded by this measurement protocol; particularly as the current study had the ability to account for the effects of objective sleep continuity and architecture upon multiple measurement indices of the CAR. Specifically, in the current study all participants fully adhered to the required sampling instructions due to the researcher supervision. As the light levels were controlled and standardised for every participant, the CAR was unaffected by variations in environmental light levels, ensuring that all CAR measurement indices were almost entirely unaffected by light input to the SCN. From a feasibility perspective, the data of three participants could not be used. As such, this protocol may be most useful as an experimental, rather than a clinical, protocol.

The results of the current study indicate that the CAR, in terms of cortisol levels at each measurement time point, and total cortisol secretion during the measurement period, is stable in a highly-controlled sleep laboratory environment. However, awakening cortisol levels and the magnitude of increase in cortisol levels show daily variations and may be sensitive to variations in daily activities. As the current measurement protocol and environment ensure that the CAR can be studied in a highly controlled manner, where circadian and methodological variables have a minimal influence upon measurement indices, this protocol can be extended to assess the function of the CAR in more detail, and also HPA axis functioning in sleep disorders. Despite potential roles in arousal, recovery or anticipation [1, 8–10, 45], the precise function of the CAR is yet to be confirmed.

## Conclusions

The CAR, in terms of cortisol levels at each time point and the total amount of cortisol secreted during the measurement period, is stable across two consecutive mornings of measurement in a highly-controlled sleep laboratory environment, when controlling for important methodological factors. However, awakening cortisol levels and the magnitude of increase show daily variations and are potentially sensitive to differences in daily activities. Additionally, the Morning 2 CAR magnitude was positively associated with the Night 1 percentage of time spent in N2 sleep. This measurement protocol can also potentially be used to examine the function of the CAR and assess HPA axis function in various sleep disorders.

## Additional files

Additional file 1:
**Cortisol levels (nmol/l) at each measurement time point (** ***n*** **= 15).** (DOCX 14 kb) Additional file 2:
**Spearman correlations between Night 1 objective measures of sleep continuity and architecture and Morning 2 cortisol awakening response indices (** ***n*** **= 15).** (DOCX 15 kb) Additional file 3:
**Spearman correlations between Night 2 objective measures of sleep continuity and architecture and Morning 3 cortisol awakening response indices (** ***n*** **= 15).** (DOCX 15 kb)

## Abbreviations

ANOVA: analysis of variance
AUC_G_: area under the curve with respect to ground
CAR: cortisol awakening response
ECG: electrocardiogram
EEG: electroencephalography
EMG: electromyogram
EOG: electrooculogram
HPA: hypothalamic-pituitary-adrenal
MnInc: mean increase
N1: stage 1 sleep
N2: stage 2 sleep
N3: stage 3 sleep
nmol/l: nanomoles per litre
NWAK: number of awakenings
PSG: polysomnography
REM: rapid eye movement
SCN: suprachiasmatic nucleus
SD: standard deviation
SE%: sleep efficiency (%)
SEM: standard error of the mean
TST: total sleep time
WASO: wake after sleep onset
