# Supplementary material for: Assessing the daily stability of the cortisol awakening response in a controlled environment
Source: BMC Psychol. 2016 Jan 28;4:3. doi: 10.1186/s40359-016-0107-6 (PMC4730747; doi:10.1186/s40359-016-0107-6)
Supplement: Additional file 1: — Cortisol levels (nmol/l) at each measurement time point ( n = 15). (DOCX 14 kb) [file 40359_2016_107_MOESM1_ESM.docx]

| Additional Table 1:  *Cortisol levels (nmol/l) at each measurement time point (n = 15).* | | | | | | | | | | |  |  |  |  |  |  |
| --- | --- | --- | --- | --- | --- | --- | --- | --- | --- | --- | --- | --- | --- | --- | --- | --- |
|  | Awakening | | +15 minutes | | +30 minutes | | +45 minutes | | +60 minutes | | Morning 2 vs  Morning 3 | | Time point comparison | | Morning × time point | |
|  | Mean | *SD* | Mean | *SD* | Mean | *SD* | *Mean* | *SD* | *Mean* | *SD* | *F* value | *p-*value | *F-value* | *p-value* | *F-value* | *p-value* |
| Morning 2 | 8.85 | 4.47 | 9.10 | 4.26 | 10.58 | 3.62 | 9.40 | 3.97 | 8.66 | 3.56 | 0.08 | 0.79 | 7.44 | <0.001 | 2.19 | 0.08 |
| Morning 3 | 6.80 | 3.61 | 8.58 | 3.71 | 10.47 | 3.98 | 10.03 | 4.33 | 10.01 | 4.08 |  |  |  |  |  |  |
|  | | | | | | |  |  |  |  |  |  |  |  |  |  |
