# Supplementary material for: Assessing the daily stability of the cortisol awakening response in a controlled environment
Source: BMC Psychol. 2016 Jan 28;4:3. doi: 10.1186/s40359-016-0107-6 (PMC4730747; doi:10.1186/s40359-016-0107-6)
Supplement: Additional file 2: — Spearman correlations between Night 1 objective measures of sleep continuity and architecture and Morning 2 cortisol awakening response indices ( n = 15). (DOCX 15 kb) [file 40359_2016_107_MOESM2_ESM.docx]

| Additional Table 2:  *Spearman correlations between Night 1 objective measures of sleep continuity and architecture and Morning 2 cortisol awakening response indices (n = 15)* | | | | | | | | | | | | | |
| --- | --- | --- | --- | --- | --- | --- | --- | --- | --- | --- | --- | --- | --- |
|  | TST | SOL | NWAK | WASO | SE (%) | Time in REM | Time in N1 (%) | Time in N2 (%) | Time in N3 (%) | Latency to REM | Latency to N1 | Latency to N2 | Latency to N3 |
| Awakening levels | 0.00 | 0.12 | -0.28 | -0.34 | 0.07 | 0.48 | -0.10 | -0.62 | 0.35 | -0.01 | 0.12 | 0.09 | 0.02 |
| AUC_G_ | 0.22 | -0.04 | 0.06 | -0.05 | 0.08 | 0.46 | -0.11 | -0.46 | 0.27 | -0.04 | -0.04 | -0.04 | -0.02 |
| MnInc | 0.34 | -0.20 | 0.19 | 0.17 | 0.16 | -0.29 | -0.03 | 0.76* | -0.55 | 0.04 | -0.20 | -0.15 | 0.03 |
| Notes: **p* < 0.0013.  *Abbreviations:* AUC_G_: area under the curve with respect to ground, MnInc: mean increase, N1: stage 1 sleep, N2: stage 2 sleep, N3: stage 3 sleep, NWAK: number of awakenings, REM: rapid eye movement sleep, SE: sleep efficiency, SOL: sleep onset latency, TST: total sleep time, WASO: wake after sleep onset. | | | | | | | | | | | | | |
